# Interpreting ultraprocessed food trial evidence: evidentiary limits, reporting practices, and policy relevance

**DOI:** 10.1016/j.ajcnut.2026.101395

**Published:** 2026-06-10

**Authors:** Jimmy Chun Yu Louie

**Affiliations:** Discipline of Dietetics, School of Health Sciences, Swinburne University of Technology, Hawthorn, Victoria, Australia

**Keywords:** ultraprocessed foods, NOVA classification, randomized controlled trials, food processing, dietary interventions, evidence interpretation, nutrition policy, causal inference

## Abstract

Ultraprocessed foods (UPFs) have become a central topic in nutrition science, with extensive observational research linking higher intake to adverse health outcomes. More recent randomized controlled trials have been presented as strengthening causal claims, yet closer examination reveals a pattern of interpretation that extends beyond what the data support. This perspective reviews 4 trials that directly compared ultraprocessed diets with minimally processed diets, examining both their findings and how those findings have been represented in scientific commentary. Across all studies, the comparison group limited the ability to isolate processing as the causal factor, as multiple dietary features differed simultaneously. Reporting consistently emphasized findings aligning with expectations of harm while downplaying neutral or contradictory evidence, including favorable clinical markers and higher adherence in some ultraprocessed conditions. Methodological limitations were also underrepresented in secondary interpretations. The current experimental evidence therefore requires cautious interpretation. Specifically, 4 actionable recommendations follow from this analysis: *1*) future trials should match intervention arms on nutrient composition so that processing effects can be disaggregated from nutritional quality effects; *2*) adherence and dropout rates should be reported as primary, not secondary, outcomes; *3*) terms such as “excess consumption” and “overeating” should be reserved for conditions of positive energy balance and weight gain; and *4*) the heterogeneity within NOVA Group 4 warrants subcategory analyses rather than class-wide causal claims. Without these changes, the evidentiary basis for UPF policy risks overstating certainty and misdirecting intervention targets.

## Introduction

The NOVA classification system [1] has gained significant traction in nutrition research and public health policy discussions, moving beyond traditional nutrient-based approaches to consider ultraprocessed foods (UPFs) by their industrial formulation, ingredient displacement, and additive load [2]. Extensive observational research has consistently linked higher UPF consumption to increased risk of obesity, cardiovascular disease, type 2 diabetes, and other chronic conditions across diverse populations worldwide [[3], [4], [5], [6]], with effect sizes that often show dose–response relationships aligned with Bradford Hill criteria [7]. The proposed mechanistic pathways are plausible: processing can alter food matrix structure [8], affect nutrient bioavailability [9,10], introduce additives with limited long-term safety data [11], and generate novel compounds through Maillard reaction [12,13].

However, as randomized controlled trials (RCTs) evidence emerges [[14], [15], [16], [17]], a tendency toward selective interpretation, prominent in the UPF literature, has shaped how these findings are communicated. Scientific commentators [[18], [19], [20]] have selectively emphasized results that appear to confirm existing beliefs while downplaying contradictory evidence, representing a threat to evidence-based nutrition science. This perspective examines the 4 published RCTs, analyzing how their findings have been reported in academic commentary and identifying specific instances where interpretation has diverged from the actual study results.

## Conceptual Framework

A shared design characteristic shapes interpretation across all 4 trials: each compares UPF diets against minimally processed food (MPF) diets. This is methodologically understandable for establishing internal validity, but it means trials inevitably compare dietary patterns that differ simultaneously across nutritional composition, palatability, food matrix, and preparation mode, making it impossible to isolate processing as the sole causal factor [21]. This constraint does not invalidate findings, but it necessitates more cautious attribution of effects specifically to the degree of processing [22,23].

NOVA Group 4 also encompasses foods with markedly different nutritional profiles and processing methods, from sugar-sweetened beverages (SSBs) and confectionery to wholegrain breads with emulsifiers and fortified plant-based alternatives [24,25]. Whether observed associations reflect uniform effects across all UPFs or are driven by specific subgroups with particularly adverse characteristics remains unresolved [25,26]. Applying mechanistic evidence derived from specific additives or processing technologies to the entire NOVA Group 4 class [[27], [28], [29]] represents an extrapolation that the current experimental evidence does not support.

## Evidence from Randomized Controlled Trials

The key features, strengths, and limitations of each trial are summarized in Table 1. Three cross-cutting interpretational problems emerge from their collective consideration.

**TABLE 1:** Summary of ultraprocessed food randomized controlled trials to date

Study	Sample size, duration, and study design	Key findings	Strengths	Limitations
NIH Metabolic Ward Study [15]	*n* = 20 28 d total (14 d per diet), no washout Randomized crossover; metabolic ward setting	- +508 kcal/d on UPF diet - Weight gain on UPF, weight loss on MPF	- Gold-standard controlled environment - Precise measurement of intake and outcomes - Established causal framework for UPF research	- Dramatic differences in food types beyond processing (ravioli vs. baked cod) - Confounding of palatability and cultural food associations - Short duration limits generalizability
Japanese Crossover Study [16]	*n* = 9 Japanese men with overweight/obesity 14 d total (7 d per diet), 2-wk washout Randomized crossover; open-label	- +813.5 kcal/d on UPF diet - 1.1 kg additional weight gain on UPF	- Provided converging evidence supporting NIH findings - Real-world dietary delivery	- Extremely small sample size - Very short intervention period - Open-label design introduces expectation bias - Magnitude of caloric differences questions ecological validity - Significant confounding from fiber differences (43.3 vs. 77.3 g designed; 35 vs. 50 g actual) with different fiber types (soluble in UPF vs. insoluble in non-UPF) affecting chewing and satiety independently of processing level
UPDATE Trial [14]	*n* = 55 16 wk total (8 wk per diet); 4-wk washout Randomized crossover; home-delivered meals	- Both diets resulted in weight loss - MPF achieved greater weight loss (−2.06% vs. −1.05%) - Better adherence to UPF diet	- Longest intervention period - Largest sample - Real-world meal delivery	- Superior adherence to UPF diet challenges negative narrative
Copenhagen Male Reproductive Health Study [17]	*n* = 43 men aged 20–35 12 wk total (3 wk per diet); 3-mo washout 2 × 2 crossover (UPF vs. unprocessed × adequate vs. excess calories)	- Increased body weight and LDL:HDL ratio with UPF independent of caloric load - Hormonal changes affecting metabolism and spermatogenesis	- Novel demonstration of UPF effects independent of caloric intake - Innovative study design separating processing from caloric effects	- Significant confounding from nutritional profile between the interventions - Sperm quality effects only "trended toward impairment" without statistical significance - 3-wk intervention shorter than sperm production cycle

Abbreviations: MPF, minimally processed food; UPF, ultraprocessed food.

First, nutritional nonequivalence is treated as processing evidence. In the Copenhagen study [17], the UPF diet provided 13.7 g saturated fat per 1000 kcal compared with 6.6 g, 51.0 g added sugar compared with 2.5 g, and only 5.0 g fiber compared with 19.5 g, where represent deficiencies relative to WHO and National Health and Medical Research Council (Australia) reference standards [[30], [31], [32]]. The large metabolic differences observed in that trial are more plausibly attributable to nutritional inadequacy than to processing per se. Yet commentary has emphasized the processing interpretation. Similarly, in the Japanese study [16], a fiber deficit of ∼15 g/d in the UPF arm, and qualitatively different fiber types, may explain the observed excess energy intake independently of processing [33].

Second, contradictory findings are systematically under-reported. The UPDATE trial [14] is the most instructive case. Both diet conditions produced weight loss; the characterization of the smaller UPF weight loss as “excess consumption” [20] misuses the term, which in clinical and research contexts denotes positive energy balance and weight gain. Neither of them occurred. Beyond weight, zero participants withdrew during UPF periods compared with 18% during MPF periods (despite all meals being provided free of charge and home-delivered), and several cardiometabolic markers (heart rate, fasting glucose, and LDL-cholesterol) were more favorable on the UPF diet. None of these findings received prominent treatment in commentary.

Third, the causal language applied to early findings has been extended beyond its evidentiary basis. The NIH metabolic ward study [15] demonstrated that ad libitum UPF consumption increased energy intake under highly controlled conditions, which is a valid and important internal finding. The diets differed substantially in palatability and food type beyond processing level alone, meaning the effect cannot be attributed solely to processing degree [25,34]. These constraints are inherent to controlled feeding trials and do not diminish the trial's contribution, but they do limit the scope of legitimate causal inference.

## Implications and Recommendations

The field requires specific methodological reforms, not just a general call for balance. Four concrete recommendations emerge from the analysis above:

1) Future trials should match intervention arms on key nutritional parameters (energy density, macronutrient ratio, fiber content and type, and micronutrient profile) so that processing effects can be disaggregated from nutritional quality effects. Without nutritional equivalence, RCT results are uninformative about the causal role of processing per se.
2) Adherence and dropout rates should be treated as primary, not secondary, outcomes in UPF feeding trials. The UPDATE trial's zero UPF dropout alongside 18% MPF dropout is directly relevant to the real-world effectiveness of dietary interventions and should be foregrounded, not omitted from commentary [14].
3) Terminological precision is required: “excess consumption,” “overeating,” and “causality” should be reserved for conditions of positive energy balance, weight gain, and randomized evidence with appropriately equivalent comparison arms. Using these terms when both intervention groups lose weight, or when diets differ on multiple nutritional dimensions, generates misleading claims about what the evidence establishes.
4) Subcategory analyses within NOVA Group 4 should become standard. Given the nutritional heterogeneity of the category, spanning SSBs, confectionery, emulsifier-containing wholegrain breads, and fortified plant-based milks [24,25,35], class-wide causal claims are unlikely to reflect uniform biological mechanisms. Research comparing NOVA Group 3 (processed) with NOVA Group 4 (ultraprocessed) foods is also needed to establish whether the processing step itself, rather than the broader dietary pattern it represents, is the active variable [36,37].

## Concluding Remarks

The 4 published UPF RCTs provide genuine and important experimental evidence; however, the gap between what each trial was designed to test and what has been claimed on its behalf is consequential. The NIH study established that ad libitum UPF consumption increases energy intake in a metabolic ward, not that processing causally drives weight gain across free-living populations. The UPDATE trial showed both UPF and MPF diets produce weight loss, with better adherence and several favorable clinical markers on the UPF arm, not that UPF diets cause excess consumption. The Copenhagen study demonstrated metabolic changes under marked nutritional nonequivalence, not that processing per se drives those changes. Progress in this field requires that scientific commentary matches the precision of the studies themselves: reporting all relevant findings, specifying what each trial can and cannot establish, and reserving causal language for conditions that support it. The 4 recommendations above, nutritionally matched designs, mandatory adherence reporting, terminological precision, and subcategory analyses, are achievable and would materially strengthen the evidence base underpinning UPF policy.

## Author contributions

The authors’ responsibilities were as follows – JCYL is the sole author and is responsible for all aspects of this work.

## Declaration of Generative AI and AI-assisted technologies in the writing process

During the preparation of this work, the author used Claude sonnet 4.6 (https://claude.ai) in May 2026 to improve the grammar and clarity of the text he drafted. After using this tool/service, the corresponding author reviewed and edited the content as needed and takes full responsibility for the content of the publication.

## Funding

The authors reported no funding received for this study.

## Conflict of interest

The author reports no conflicts of interest.
